# Artificial Intelligence Meets Marine Ecotoxicology: Applying Deep Learning to Bio-Optical Data from Marine Diatoms Exposed to Legacy and Emerging Contaminants

**DOI:** 10.3390/biology10090932

**Published:** 2021-09-18

**Authors:** Nuno M. Rodrigues, João E. Batista, Pedro Mariano, Vanessa Fonseca, Bernardo Duarte, Sara Silva

**Affiliations:** 1 LASIGE, Faculty of Sciences, University of Lisbon, Campo Grande, 1749-016 Lisbon, Portugal; jebatista@fc.ul.pt (J.E.B.); sara@fc.ul.pt (S.S.); 2 Biosystems and Integrative Sciences Institute (BioISI), Faculty of Sciences, University of Lisbon, Campo Grande, 1749-016 Lisbon, Portugal; plmariano@fc.ul.pt; 3 MARE—Marine and Environmental Sciences Center, Faculty of Sciences, University of Lisbon, Campo Grande, 1749-016 Lisbon, Portugal; vffonseca@fc.ul.pt (V.F.); baduarte@fc.ul.pt (B.D.); 4 Departamento de Biologia Animal, Faculty of Sciences, University of Lisbon, Campo Grande, 1749-016 Lisbon, Portugal; 5 Departamento de Biologia Vegetal, Faculty of Sciences, University of Lisbon, Campo Grande, 1749-016 Lisbon, Portugal

**Keywords:** artificial intelligence, machine learning, deep learning, time series classification, estuarine systems, eutrophication, water contamination

## Abstract

**Simple Summary:**

Our work is motivated by the increasing production of chemicals with environmentally harmful effects to our aquatic ecosystems. We show that it is possible to detect and distinguish the presence of several different emerging contaminants, using the photochemical responses of a microalgae species, which is among the most abundant phytoplankton group in the oceans. We use several machine learning and deep learning models that operate on chlorophyll fluorescence induction curves, which are composed of fluorescence values taken at different time steps from the microalgae exposure trials, achieving up to 97.65% accuracy when predicting the type of contaminant, and up to 100% in several cases when predicting the exposure concentration. Our results show the combination of these models with the fluorescence induction curves creates a powerful tool for ecotoxicity assessment, capable of classifying model organisms for their contaminant exposure, both in terms of type and concentration, opening new doors for toxicophenomics developments.

**Abstract:**

Over recent decades, the world has experienced the adverse consequences of uncontrolled development of multiple human activities. In recent years, the total production of chemicals has been composed of environmentally harmful compounds, the majority of which have significant environmental impacts. These emerging contaminants (ECs) include a wide range of man-made chemicals (such as pesticides, cosmetics, personal and household care products, pharmaceuticals), which are of worldwide use. Among these, several ECs raised concerns regarding their ecotoxicological effects and how to assess them efficiently. This is of particular interest if marine diatoms are considered as potential target species, due to their widespread distribution, being the most abundant phytoplankton group in the oceans, and also being responsible for key ecological roles. Bio-optical ecotoxicity methods appear as reliable, fast, and high-throughput screening (HTS) techniques, providing large datasets with biological relevance on the mode of action of these ECs in phototrophic organisms, such as diatoms. However, from the large datasets produced, only a small amount of data are normally extracted for physiological evaluation, leaving out a large amount of information on the ECs exposure. In the present paper, we use all the available information and evaluate the application of several machine learning and deep learning algorithms to predict the exposure of model organisms to different ECs under different doses, using a model marine diatom (*Phaeodactylum tricornutum*) as a test organism. The results show that 2D convolutional neural networks are the best method to predict the type of EC to which the cultures were exposed, achieving a median accuracy of 97.65%, while Rocket is the best at predicting which concentration the cultures were subjected to, achieving a median accuracy of 100%.

## 1. Introduction

In recent decades, there is an evident increase in the development of uncontrolled human activity [1]. The relationship between increased human population density and environmental changes in coastal regions is well known. Coastal waters are the ultimate sink of sewage and other by-products of human activities. The EU’s Task Group for the Marine Strategy Framework Directive (MSFD) implementation recommended that monitoring programs covering the chemical contaminants concentrations should also integrate biological measurements of the effects of the contaminants on marine organisms [2]. The combination of conventional and newer, effect-based methodologies, with the assessment of environmental contaminant concentrations, provides a powerful and comprehensive approach [2]. It is also striking that the pace of chemical discovery is growing rapidly, with the Chemicals Abstracts Service (CAS REGISTRY) reporting in May 2011 the registration of the 60th million chemical substance, and the 50th million substance registration in only 2009, highlighting the continued acceleration of synthetic chemical innovation [3]. Among these, several contaminants have raised concerns due to their ecotoxicological effects and how to assess them efficiently. For example, pesticides continue to be detected in surface and groundwater [4], pharmaceuticals, concentrated in wastewaters discharged from households and medical facilities are also present in coastal areas [5] and personal care products are being detected in phytoplankton cells even at remote locations [4], highlighting their environmental persistence.

At the basis of most marine systems are phototrophs, cycling solar energy, and soaking carbon, fueling the trophic web. Any disturbance at this level has inevitable impacts on the whole marine ecosystem. Contaminants toxic effects are known to have severe and specific influence on these organisms, especially impairing their photosynthetic metabolism [6,7,8,9,10]. Having both biochemical and biophysical components allows the photosynthetic mechanisms to be addressed remotely [11]. Pulse amplitude modulated (PAM) fluorometry emerges as a potential non-invasive high-throughput screening (HTS) tool [9,10]. These techniques use optical signatures as a proxy of the phototroph physiology, allowing for detection of disturbances at the primary productivity level [12], to efficiently evaluate contaminants’ effects with a dose-related response [13], and have been integrated into numerical indexes, easily used by stakeholders [12,14]. The integrated repeated measures, over time, without organism scarification is another key benefit of this approach. Phytoplankton is the first compartment to be affected by contaminants. As small (0.2–200 um), single or chain-forming, cells suspended in water with very high surface:volume ratios, they respond quickly to suspended toxicants with high uptake rates, being used as pollution indicator species [15].

These facts are the cornerstone of the pioneering research field of toxicophenomics, which merges plant phenomics, measuring traits, such as plant growth and performance using non-invasive technologies, with ecotoxicology, shifting the use of these phenotyping tools to address contaminant induced stress in autotrophs. Optical techniques to disclose different groups of samples exposed to different degrees of contamination has been applied in marine phototrophs with a high degree of efficiency, in both ecotoxicological trials and under field conditions [6,8,16], and using communities as meta-organisms instead of single species [17]. The application of these bio-optical technologies produces large data packages with physiological interest, regarding the effects of a given compound in an autotrophic organism, and also with a high volume of data points that can be used by machine learning techniques, to produce classifiers of the toxicity degree to which organisms are exposed [18]. Consequently, the increase in big data from HTS systems needs efficient data processing and analytical pipelines. These bio-optical data have been successfully used in the past as biomarkers for contaminant exposure with a high degree of efficiency, although using only less than 10% of the information provided by these optical techniques. These optical biomarkers have been used from field to experimental trials using a wide array of organisms from microalgae to higher plants [9,10,14]. Conventional data analysis pipelines typically involve computer vision tasks (e.g., wheat head counting using object detection), which are addressed through the development of signal processing or machine learning (ML) algorithms [19]. When exposed to anthropogenic contaminants the typical chlorophyll *a* induction curves suffer alterations, not only on their intensity values but also on their shape [9]. These curves are composed of more than 400 data points referent to the chlorophyll *a* fluorescence echo of the organism derived from its photochemical fitness, but only some key points are effectively used for photochemical variable calculation, discarding a high number of data points that can be potentially used for high-throughput screening of the test organisms [9]. Contaminant exposure affects not only the specific time points used for photochemical variable calculation but also the remaining curve data points [9]. Such a large dataset is, therefore, overlooked, despite its potential to be analyzed by machine learning techniques, in order to disclose at a fine-scale resolution, the changes provoked by contaminant exposure in all the chlorophyll *a* induction curves data points, with a sensitivity that would escape the manual and traditional human analysis.

In this context, the aim of this paper is to evaluate the performance of several machine learning and deep learning methods in predicting the exposure of model organisms to different emerging contaminants (EC) under different exogenous concentrations, based on the complete set of fluorescence data as a potential set of biomarkers of contaminant exposure. This combination of machine learning with non-conventional biomarkers, intent to disentangle the potential of not only whole fluorescence profiles as descriptors (biomarkers) of contaminant exposure but also to address the power of machine learning in disentangling slight and unperceivable features that can be later used for automatic classification of marine diatom cultures exposed to a wide array of contaminants in ecotoxicological trials. For this purpose, a model ecotoxicological organism was used as a test organism, with marine diatom cultures of *Phaeodactylum tricornutum* subjected to thirteen emerging contaminants at various exposure concentrations. The resulting chlorophyll fluorescence induction curves are used as time series data, along with pre-defined endpoints, were used to address two tasks: (i) identifying which of the thirteen contaminants considered was applied, and (ii) at which exposure concentration. To address both points, we used five different machine learning and deep learning algorithms:

- **Random Forests:** This classifier is an ensemble algorithm that uses the majority vote of a set of decision trees to make predictions;
- **XGBoost:** This classifier is a state-of-the-art ensemble algorithm that uses a set of decision trees optimized with gradient boosting in order to minimize errors;
- **ROCKET:** A recent state-of-the-art time series classification algorithm that uses random convolution kernels to increase the dimensionality of a dataset and, together with a simple regression classifier (commonly Ridge);
- **Neural Networks:** Deep learning models based on the original multi layer perceptron. These networks have a large amount and variety of layers and are trained by means of gradient descent. Although for regular artificial neural networks (ANN) the feature engineering part is done *a priori*, convolutional neural networks (CNN) perform this step using convolutional and pooling layers. CNNs can be used for multi-dimensional data, so they can process 1D, 2D, or 3D.

In this work, the 2D CNNs have the particularity of using as input data the images of the plotted time series data, instead of the numeric values.

## 2. Materials and Methods

### 2.1. Ecotoxicological Assays

*Phaeodactylum tricornutum* Bohlin (*Bacillariophyceae*; strain IO 108-01, Instituto Português do Mar e da Atmosfera (IPMA)) axenic cell cultures (maintained under asexual reproduction conditions) were grown in f/2 medium [20], under constant aeration in a phytoclimatic chamber, at 18 °C, programmed with a 14/10 h day/night photoperiod (RGB 1:1:1, maximum PAR 80 μmol photons m-2 s-1), a sinusoidal function to mimic sunrise and sunset, and light intensity at noon, set to replicate a natural light environment [21]. Cultures are visually inspected periodically, under the microscope to ensure their axenic state. Exposure trials were conducted according to the Organization for Economic Cooperation and Development (OECD) recommendations for algae assays (OECD, 2011), with minor adaptations, and the suggested initial cell density for microalgae cells with comparable dimensions to ı (initial cell density $=2.7\times 10^{5}$ cells mL${}^{-1}$). According to OECD guidelines, carbon was provided to cultures through aeration with ambient air. Exposure time was reduced to 48 h since in previous studies was observed that after 72 h the cultures enter the stationary phase and, thus, exhibit ageing effects that can mask the exposure trial [21]. Exposure conditions in terms of nutrient composition, light, and temperature environment were kept as those for culture maintenance. Forty-eight hours after inoculation (endpoint), cells were exposed to the target concentrations of the selected emerging contaminants (diclofenac, ibuprofen, propanol, fluoxetine, glyphosate, sodium dodecyl sulphate (SDS), triclosan, copper engineered nanoparticles (CuO), zinc engineered nanoparticles (ZnO), and titanium engineered nanoparticles (TiO)) and legacy contaminants (dissolved ionic copper (CuSO${}_{4}$), dissolved ionic Zn (ZnSO${}_{4}$), and dissolved ionic titanium (TiO${}_{2}$)). Exposure occurred for 48 h to ensure it covered the exponential growth phase [21,22,23]. Target concentrations were selected aiming to cover a concentration gradient reflecting not only the detected environmental concentrations found in the literature, but also concentrations known to have significant biological effects in *P. tricornutum*. As observed in previous works [21,22,23] this is a fast-growing strain, hence the exposure period was reduced from 72 h to 48 h to avoid cell ageing processes, that could occur in the stationary phase beyond the 48 h time point. All manipulations were executed within a laminar flow hood chamber, ensuring standard aseptic conditions. At the end of the exposure period, 1 mL of culture (per replicate) was used for bio-optical assessment, via chlorophyll-a pulse amplitude modulated (PAM) fluorometry (FluorPen FP100, Photo System Instruments, Brno, Czech Republic). Cell subsamples for bio-optical assessment were acclimated for 15 min in the dark and chlorophyll transient light curves (Kautsky plots) were generated using the preprogrammed OJIP protocol, according to [23].

Each sample corresponds to a chlorophyll fluorescence induction curve and contains the fluorescence values taken at different time steps from a model diatom used in ecotoxicology (*Phaeodactylum tricornutum*) exposed to 13 different emerging contaminants at different concentrations, following the international standards for ecotoxicological assays. Each curve is composed of 458 measurements per sample, taken in intervals of 10 milliseconds each. For each contaminant and concentration, 30 independent replicates were obtained, totaling 1950 samples. Table 1 contains some details regarding this dataset, including the identification of the contaminants and different concentrations used, and the distribution of samples between contaminants and concentrations. Further details on the techniques and protocols used for obtaining the data can be found in [6,7,8,16,24]. Cell density was evaluated in all experimental reactors at the end of the exposure trials and the percentage of growth inhibition towards the respective control condition was calculated, as well as the half-maximal inhibitory concentration (IC50), according to the OECD (2011) guidelines.

The same dataset is used differently for the two tasks we address. For the task of identifying the contaminant, we consider 13 classes, each corresponding to one EC regardless of its concentration, therefore 120/150/180 samples per class, depending on the EC (see Table 1). Additionally, control test units (absence of tested compound) were also performed so that it was possible to disclose the effects of the EC and its concentrations in the physiology of the cultures under the same conditions as the test bioreactors. The task of identifying the EC concentration is subdivided into 13 subtasks, each dealing with only one EC. Each of these subtasks considers 3–5 classes, depending on the EC, where each class contains exactly 30 samples, each corresponding to a specific concentration (Table 1). In order to disentangle with a higher resolution the effects of each EC under each dose, an extended analysis of the fluorescence transients was done by calculating the difference in relative variable fluorescence towards the respective control units, allowing this way to have variation towards the control and represented as ΔV curves (expressed as V=f(t)), i.e., subtracting fluorescence values of the controls of transients normalized between:

(1) $F_{o}$ and $F_{P}$$\;(F_{M})$, i.e., $V_{t}=\left(F_{t}-F_{o}\right)/\left(F_{P}-F_{o}\right),\Delta V_{t}=\left(V_{t,treatment}-V_{t,control}\right)$;

(2) $\Delta W_{K}=\left(W_{K,treatment}-W_{K,control}\right)$ [25].

### 2.2. Methods

We use a set of different machine learning and deep learning algorithms to perform classification. In total, we test five algorithms, including Random Forests (RF) [26], XGBoost (XGB) [27], Rocket [28], deep artificial neural networks (ANN) and convolutional neural networks (CNN) [29].

Regarding the CNNs, we test three different approaches: 1D, where the input is the one-dimensional induction curves; 2D, where we use image representations of the induction curves; and 2D Log, like 2D but with a logarithmic time axis. Regarding the 2D CNNs, for the remainder of this work, they will be designated simply as CNNs, and the methodology used to transform the induction curves into images is described in more detail in [18].

### 2.3. Hardware and Software

All experiments were performed on a Windows 10 machine with one NVIDIA 2080 TI GPU with 11GB of RAM. All models were implemented in Python 3.6 using the following packages: Scikit-learn (0.23.1), XGBoost (1.1.1), and Tensorfow (2.3) [30,31].

### 2.4. Architecture and Parameters

This section provides technical details regarding the implementation and parameters of the used methods and is intended to aid in the reproducibility of the experiments.

For both RF and XGB trees, we perform an independent grid search per run, for the optimal values of their parameters. Regarding RF, we grid search for the following parameters: *criterion* {gini,entropy}, *No. estimators* {5,10,15,20} and *maximum depth*{2,5,7,9}. As for XGB trees, we grid search for the following parameters: *maximum depth* {2,5,7,9}, *No. estimators* {50,100,200}, *learning rate* {0.1,0.01,0.001}, and *tree method* {auto,exact,approx,hist}.

**Figure 1:**
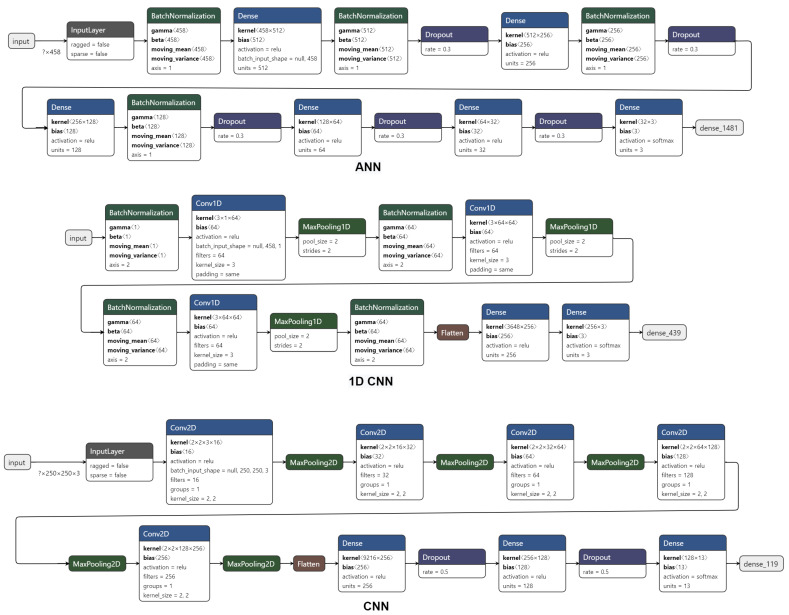
Architectures of the different deep learning models used in this work. The first network corresponds to the ANN used, it is composed by a set of BatchNormalization, Dense (fully connected), and Dropout layers. The second corresponds to the 1D CNN, composed by a set of BatchNormalization, 1D Convolution, and 1D MaxPooling layers, followed by a flatten operation to reduce the dimensionality of the data in order to pass it to the two dense layers that serve as a classifier. The last network corresponds to our 2D CNN, composed by 2D Convolution, 2D MaxPooling, a flatten and both Dense and Dropout layers. Each layer from models has its parameters discretized inside the corresponding box.

For both ANNs and CNNs, we use a simple and shallow architecture. The ANNs (These ANNs are in essence multilayer perceptrons (MLPs) with normalization and dropout.) are composed of four batch normalization layers, six fully connected layers, and five dropout layers (for the dropout layer we performed several initial tests with different rates, and since there was no significant difference, decided to use the standard rate value of 0.7). The 1D CNNs are composed of four batch normalization layers, three interspersed convolutional and max-pooling layers, and two fully connected layers. Lastly, the CNNs are composed of five interspersed convolutional and max-pooling layers, and three fully connected layers with two dropouts in between. These architectures, as well as the hyperparameters, can be seen in Figure 1. No form of grid-search or Bayesian hyperparameter and topology optimization was performed for these models, all values being derived from a small set of manual tests. Regarding weight initialization, we decided to use the Xavier/Glorot to avoid having the gradient vanishing or exploding, and every batch was normalized to have a mean of zero and a standard deviation of one (BatchNormalization layers) so the training was faster and to help improve generalization. For the optimizer, we use ADAM with a learning rate of 0.001.

### 2.5. Experimental Setup

For both tasks, we follow the same methodology regarding the splitting of the data. In each trial, we randomly select 20% of the data to be our test set, to validate the models, and the remaining 80% is used for training. We perform 30 independent runs per method, so for each task/subtask we obtain a sample of 30 results that can be statistically analyzed and compared.

The choice of the number of epochs to run with the deep learning methods was based on a set of trials. Performing increments of 50 epochs we analyzed the convergence of the training loss of the networks and stopped adding epochs once the loss appeared to have stabilized. Based on these trials, we established that both CNNs run for 300 and 100 epochs on the first and second task, respectively, and ANN and 1D CNN run for 500 epochs on both tasks.

## 3. Results

In the present work, the authors aim to study the applicability of the obtained fluorescence data in automatic machine-learning-based systems, for ecotoxicological classification of marine diatoms-based exposure trials, and, thus, the physiological responses of the cells will not be discussed in the present work, as this is out of the scope of the present work. The physiological effects of each contaminant are discussed elsewhere [6,7,8,16,23,24]. Nevertheless, and in order to better understand the magnitude of the ecotoxicological effects observed in the cultures, relative growth inhibition and half-maximal inhibitory concentrations were also determined as proxy measures of the toxicity effects observed in the cultures, that are unequivocally connected with the photochemical results (Table 2). Although the octanol–water partition coefficients have similar values among tested substances, the *P. tricornutum* exposures to these xenobiotics led to very different responses in terms of growth inhibition, a process that is intrinsically connected with the photochemical processes (Table 2).

We report our results in tables, boxplots, spider plots, and heatmaps. Accuracy values are reported as proportions (0–1) in the tables and plots, but for convenience, we also use them as percentages (0–100) in the text. When presenting median results of the 30 runs, we determine the statistical significance of the observed differences with the non-parametric Kruskal–Wallis test. Whenever we refer to better and best or worse and worst results, it means the *p*-value of the statistical test was <0.01. All the *p*-values are presented in tables in Appendix A.

**Figure 2:**
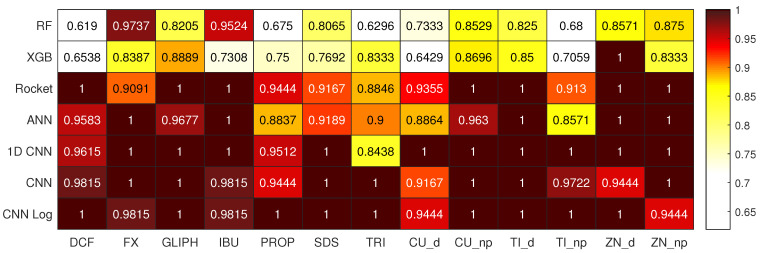
Heatmap depicting the accuracy per class of each best performing model when predicting the different types of ECs.

**Figure 3:**
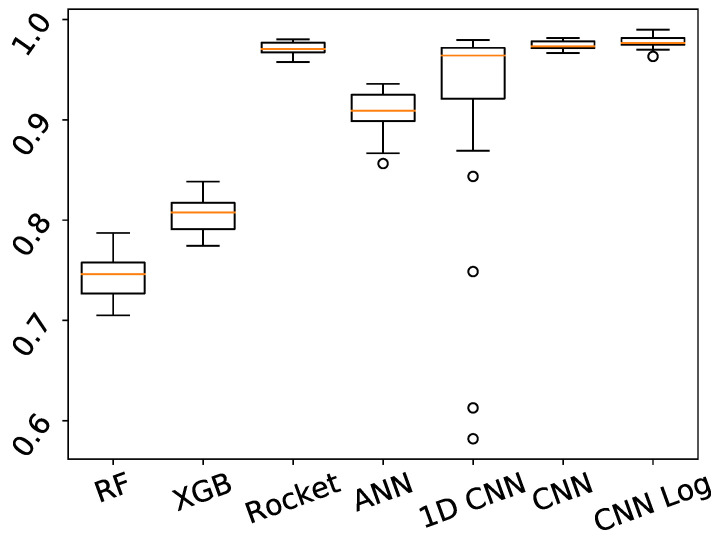
Boxplot showing the distribution of accuracy obtained by each method on the test set, in 30 independent runs of predicting the EC.

For the task of predicting the type of EC, the best results are obtained by the CNN Log, with a median accuracy of 97.65% on the test set, as detailed in Table 3. Observing the results of the best CNN Log model on the heatmap of Figure 2, we see that the vast majority of ECs are correctly classified with accuracy values of 100%, with Cu_d and Zn_np having the lowest accuracy values of 94.44%. The best models of the other deep learning methods also achieve very good results, as well as Rocket, but not so consistently in all the classes. For example, 1D CNN achieves accuracy 100% in even more classes than CNN Log, but reaches only 84.38% in one of the classes (TRI). Looking at the boxplot of Figure 3, we see that 1D CNN indeed shows a very high dispersion of results, revealing to be a less reliable method despite its high median accuracy. ANN also reveals a higher dispersion than Rocket or the CNNs. Regarding RF and XGB trees, their overall accuracy is much lower than the others in terms of median and best results.

**Figure 4:**
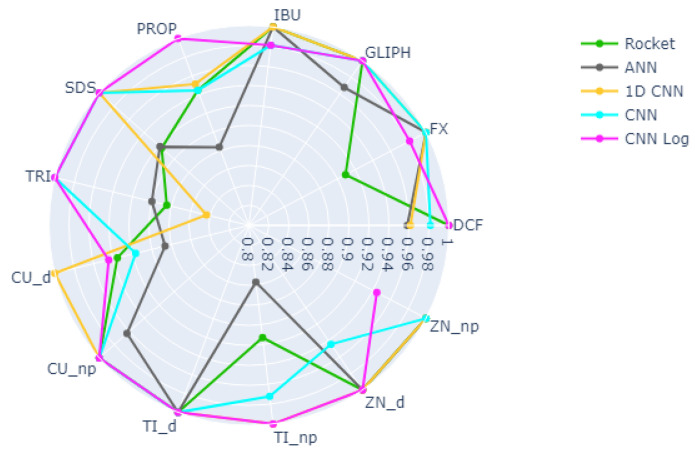
Spider plot depicting the accuracy per class of each best performing model when predicting the different types of ECs.

The spider plot of Figure 4 contains the same data as the heatmap of Figure 2, represented in a different way and omitting the RF and XGB results since these two clearly cannot compete with the other methods. The advantage of having these two plots for the same data are that while it is easier to view the large differences between the models on the spider plots, the heatmaps make it easier to compare the concrete values of the accuracy per class given that the color scheme was chosen to emphasize the small yet relevant differences that can not be seen in the spider plots. Here we can easily verify which ECs are easier or harder to predict and observe the consistency or disparity of results obtained by the different models for each EC. We observe that ECs, such as GLIPH, IBU, Cu_np, and Ti_d, are always very well classified, whereas PROP, TRI, Cu_d, and Ti_np represent a challenge for most models.

Regarding the task of predicting the concentrations of the different ECs, the scenario changes. Table 4 shows that Rocket is the superior method, achieving the best median test accuracy in all but one EC, followed by ANN which surpasses Rocket in one EC and shares the best results in 7 other ECs, and then by 1D CNN that shares 5 of the best results with Rocket and ANN. CNN and CNN Log only achieve one best result each. Regarding RF and XGB trees, although they do not claim any best results, on this task they do not perform so much worse than the other methods. SDS is the hardest EC to predict the concentration, probably because even on a small amount it simply kills the microalgae. Zn_np is another EC where predicting the concentration is not so easy.

**Figure 5:**
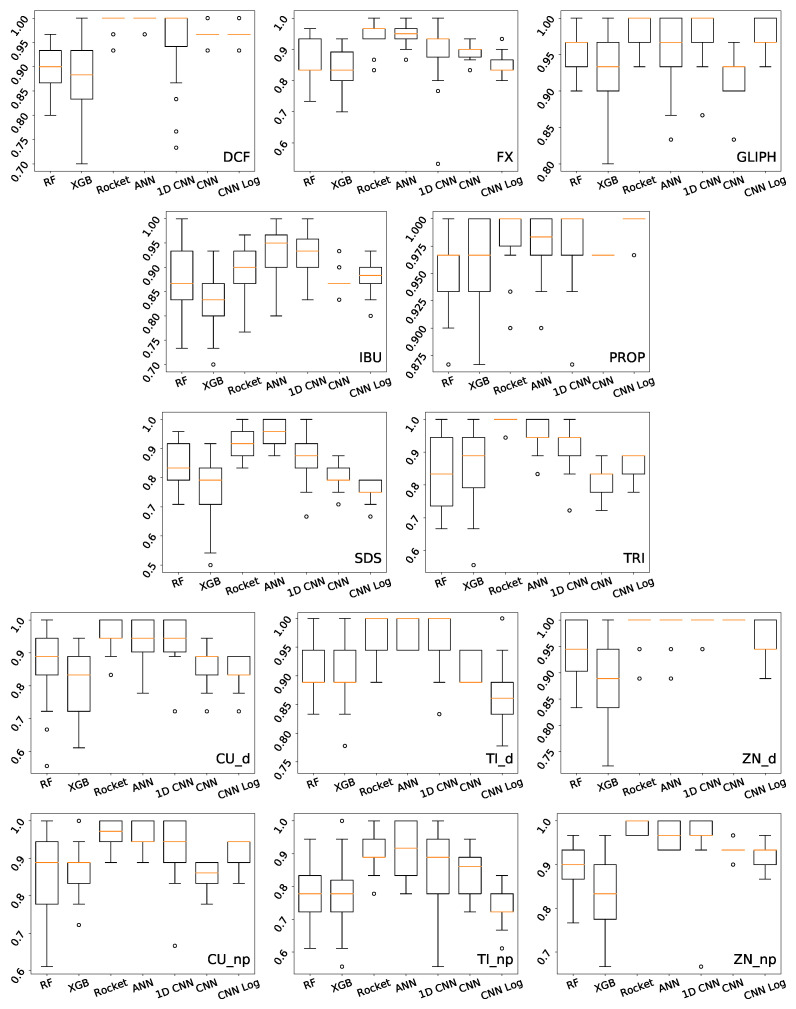
Boxplots showing the distribution of accuracy obtained by each method on the test set, in 30 independent runs of predicting the concentration of each given EC.

**Figure 6:**
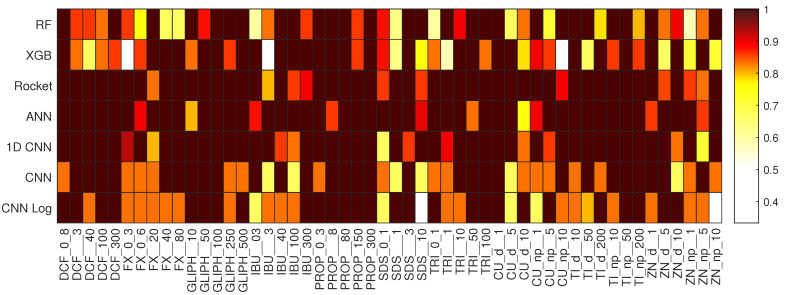
Heatmap depicting the accuracy per class of the models when predicting the different concentrations of the different ECs. This heatmap is a concatenation of 13 smaller ones. The *x*-axis lists all the ECs at different concentrations (see Table 1).

**Figure 7:**
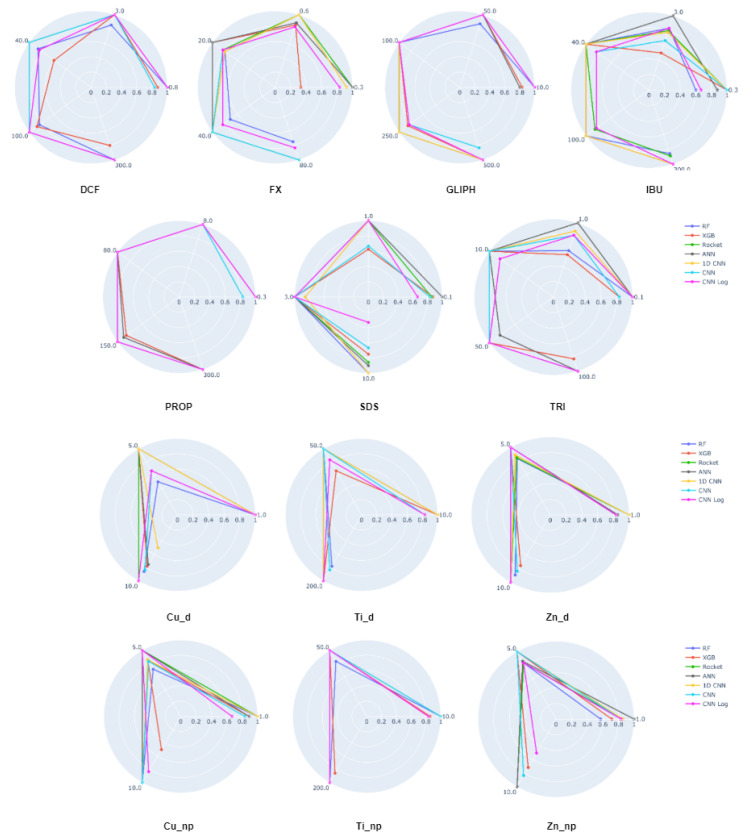
Spider plots depicting the accuracy per class of each model when predicting the different concentrations of an EC. Each vertex within the circle corresponds to the concentration of that EC.

Looking at the boxplots of Figure 5, for most ECs and methods we observe a higher dispersion of accuracy values than what we saw on the first task. Although on the first task the median accuracy was very close to the best result, here it is seldom the case. For this reason, the heatmap and spider plots that follow report the results of the models that achieve median, not best, results. The heatmap in Figure 6 and the spider plots in Figure 7 show the accuracy per concentration for all 13 ECs. The large heatmap is a concatenation of 13 smaller parts. Looking at both types of plots, we observe a large disparity of behaviors. Some classifiers are generally superior to others, but the performance of each method depends not only on the EC but also on its concentration, with no clear relationship between the concentration and the accuracy.

## 4. Discussion

The results reported above reveal that from the first task (predicting the EC) to the second task (predicting the concentration of the EC) the ranking of best methods changes. This seems to have been caused by a relative failure of the CNNs on the second task, after being so successful on the first one.

Several hypotheses may be advanced regarding this change: the normalization of the data, which is performed only for the second task (Section 2.5); the strong reduction in the number of classes (from 13 on the first task to 3–5 on the second); the strong reduction in the number of samples available in each class (from 120–180 on the first task to only 30 on the second). In order to test whether the culprit is the normalization of the data, we have performed experiments with non-normalized data for the second task. However, with this setting the CNNs obtained worse results than with normalization, thus discarding it as the cause for their failure.

Regarding the reduction in the number of classes, although it is normally expected that fewer classes mean easier problems, the labels used for the second task are not a subset of the first, but rather a refinement of each of the original labels. This defines a different problem, one that may be more difficult than the first one, even after splitting it into 13 independent subproblems. In order to check whether the set of 13 subproblems represent an easier or harder challenge than the one problem of the first task, we compare the results of predicting the EC (Table 3) with the median results of predicting the concentrations for each of the ECs (last row of Table 4). On the training set, all the methods do better on the median of the 13 problems of predicting concentrations than on the one problem of predicting the EC. In fact, not only the median is better, but also the single results of each of the 13 problems, most of them a perfect accuracy of 100%. Rocket is the only method reporting a single case where the training accuracy is higher when predicting the EC (99.91%) than when predicting the concentration of one of the ECs (IBU, 97.08%). Additionally, the CNNs do better on all the problems of the second task than on the one problem of the first task. Therefore, the learning of the training set is clearly easier on the second task. As for generalization on the test set, some methods are better on the second task (RF, XGB, Rocket, ANN) while others are worse (1D CNN, CNN, CNN Log), and this is what changes the ranking of the methods.

We investigated what causes the relative lack of generalization ability in 1D CNN and both CNNs, with a clear hypothesis in mind. One cause might be because there are not enough data to further split the training set into a validation set, which would allow us to implement an early stopping training criteria for the models. This is aggravated by the fact that these methods learn the training data very easily, and indeed we confirmed that there is a moderate amount of over-fitting. Assuming the amount of data was larger than 30 samples per class, it is possible we could have made better choices regarding the number of epochs to run, and the convolutional DL approaches would be expected to perform much better than they did on the second task. DL is indeed known to require large amounts of data to achieve a good performance, so this finding is actually not surprising, and strongly suggests the need for data augmentation.

One interesting result we obtained is related to the performance obtained by the CNNs that use logarithmic time data (CNN Log) and the CNNs that use time steps (CNN). It is common to use logarithmic scales of the temporal data for visualizing the Kautsky curves, but, of course, most ML methods only use the values of the time series for performing the classification, regardless of how they would be visualized. However, both CNN and CNN Log rely on plots of the data, and, therefore, they “see” the data differently depending on the *x*-axis, and we have observed that using a logarithmic scale is not always the best option. Although it proved to be significantly better on the first task (*p*-values of the statistical test in Appendix A), on the second task we have the opposite scenario, where the regular CNN is more often significantly better than CNN Log. This suggests that, when performing the necessary data augmentation for improving the performance of the CNNs, one easy way to implement it is to use both regular and logarithmic data.

## 5. Conclusions and Future Work

We evaluated the performance of several machine learning and deep learning methods in two different tasks of predicting the exposure of a model marine diatom (*Phaeodactylum tricornutum*) to different emerging contaminants under different doses, using only the chlorophyll fluorescence induction curves as data. The first task was to identify which of the 13 contaminants are detected, while the second task was to identify the concentration of the contaminant. We used five different machine learning and deep learning algorithms on both tasks, including Random Forests, XGBoost trees, Rocket, and several deep neural architectures, including 1D and 2D convolutional neural networks (CNNs). The 2D CNNs had the particularity of using as input data the plots of the time series data, instead of the numeric values. One of these methods (CNN Log) was given the plots with a logarithmic *x*-axis, as it is normally visualized, while the other used a linear scale with equally spaced time points (CNN). On the first task, the best results were obtained by CNN Log, with a median test accuracy of 97.65% over 30 independent runs, closely followed by other deep learning approaches and also the Rocket method. On the second task, the best method was Rocket and other deep learning approaches, while the 2D CNNs performed comparatively bad. Whereas Rocket proved to be a reliable method for both tasks, we analyzed the reasons for the relative failure of the 2D CNNs on the second task and concluded that it was caused by the occurrence of moderate over-fitting, probably due to an insufficient amount of training data.

Thus, the combination of machine learning algorithms with fluorescence HTS data resultant from fluorescence induction curves appears as a powerful tool for ecotoxicity assessment fast large data package evaluation aiming to classify model organisms, such as diatoms, for their contaminant exposure both in terms of type and concentration, opening new doors for toxicophenomics developments.

Markedly, future work should also progress to test contaminant mixtures, as well as complex phytoplankton communities, in order to produce models with broader ecological meaning and providing a better assessment of contaminants exposure in natural environments, despite current international ecotoxicological and risk assessment guidelines largely focus on individual chemicals of concern, which are simpler to address although unlikely to occur in natural environments.

In the future, we also plan to perform data augmentation by training the models using both regular and logarithmic data, as both of them provided good results without one proving to be overall better than the other. Another thing we intend to do in the future is to test and develop models to detect which chemical form, either ionic solution or engineered nanoparticles, is the EC, by comparing the induction curves of the same concentration produced by both. Beyond the bioinformatics point of view, the present work also highlights the potential of the application of these machine learning algorithms to assist ecotoxicological evaluation of model diatom cultures exposed to a myriad of contaminants. The proposed analysis pipelines can be integrated into an automatic bio-optical assessment system fed by fluorescence sensors producing accurate classifications of the toxicity degree to which the cells are subjected to, and thus reducing operator-associated artifacts and errors. This opens a new door for ecotoxicology not only reinforcing the potential of bio-optical features to be used as biomarkers of contaminant exposure but also introducing machine learning methodologies as tools to assist the ecotoxicological classifications which are on the basis of key assessments, such as Ecological Risk Assessment (ERA), crucial for impact and biomonitoring studies. Moreover, the data analysis pipeline here proposed, can be easily transposed to other autotrophic organisms, subjected to different stress/xenobiotic conditions, once calibrated and validated against known biochemical or morphological descriptors of stress.

## Figures and Tables

**Table 1:** OPTOX dataset summary, containing the contaminants, the different concentration values and the distribution of samples among them. The first section of the table includes pharma, pesticides, and personal care products, while the second section includes metals and nanoparticles.

EC		Concentrations (μg L${}^{-1}$)	#Samples	#Samples per Concentration
Diclofenac	dcf	[0,0.8,3,40,100,300]	180	30
Fluoxetine	fx	[0,0.3,0.6,20,40,80]	180	30
Glyphosate	gliph	[0,10,50,100,250,500]	180	30
Ibuprofen	ibu	[0,0.3,3,40,100,300]	180	30
Propranolol	prop	[0,0.3,8,80,150,300]	180	30
Sodium Dodecyl Sulphate	sds	[0,0.1,1,3,10]	150	30
Triclosan	tri	[0,0.1,1,10,50,100]	180	30
Dissolved Ionic Copper	Cu_d_	[0,1,5,10]	120	30
Copper Engineered Nanoparticles	Cu_np_			
Dissolved Ionic Titanium	Ti_d_	[0,10,50,200]	120	30
Titanium Engineered Nanoparticles	Ti_np_			
Dissolved Ionic Zinc	Zn_d_	[0,1,5,10]	120	30
Zinc Engineered Nanoparticles	Zn_np_			

**Table 2:** Octanol–water partition coefficients (log KOW; NA—not applicable) and concentrations applied of the tested compounds, and respective growth inhibition (%, negative values indicate growth inhibition) and half-maximal inhibitory concentration (IC${}_{50}$) assessed for each exposure trial set.

Exposure	log K (OW) (OECD 107)	Concentration (μg L${}^{-1}$)	Growth Inhibition (%)	IC${}_{50}$ (μg L${}^{-1}$)
Diclofenac	1.9	0	0	318.9
		0.8	−8.5	
		3	−14.9	
		40	−34.9	
		100	−38.9	
		300	−42.8	
Propranolol	3.12	0	0	194.6
		0.3	−19.37	
		8	1.7	
		80	−31	
		150	−68.6	
		300	−56.1	
Fluoxetine	4.65	0	0	47.3
		0.3	−7.42	
		0.6	−10.1	
		20	−24.8	
		40	−38.8	
		80	−82.8	
Ibuprofen	2.48	0	0	350.6
		0.8	−17.1	
		3	−20.1	
		40	−18.2	
		100	−37.7	
		300	−40	
Glyphosate	−1.6	0	0	225.9
		10	0.92	
		50	−21	
		100	−28	
		250	−75.5	
		500	−89.2	
SDS	−2.03	0	0	11.4
		0.1	−15.5	
		1	−21.1	
		3	−24.6	
		10	−43.8	
Triclosan	4.76	0	0	691.7
		0.1	−0.55	
		1	−1.41	
		10	−1.6	
		100	−2.39	
Dissolved Ionic Copper	NA	0	0	14.6
		1	−12.2	
		5	−24.1	
		10	−33.6	
Copper Engineered Nanoparticles	NA	0	0	8.31
		1	−21	
		5	−33.1	
		10	−57.7	
Dissolved Ionic Titanium	NA	0	0	577.5
		10	−5.15	
		50	−11.9	
		200	−18	
Titanium Engineered Nanoparticles	NA	0	0	634.3
		10	12.3	
		50	0.47	
		200	−7.46	
Dissolved Ionic Zinc	NA	0	0	73.9
		1	−7.56	
		5	−6.51	
		10	−8.37	
Zinc Engineered Nanoparticles	NA	0	0	88.4
		1	3.39	
		5	−4.27	
		10	−7.22	

**Table 3:** Median overall accuracy obtained by the different methods when predicting the different types of ECs on the training and test set. The best test result is represented in green.

	Train	Test
Random Forests	0.9625	0.7461
XGBoost	0.9983	0.8076
Rocket	0.9991	0.9705
ANN	0.8493	0.9089
1D CNN	0.9993	0.9641
CNN	0.9815	0.9732
CNN Log	0.9807	0.9765

**Table 4:** Median overall accuracy obtained by the different methods when predicting the different concentrations of the ECs on the training and test set. The best test results for each EC are represented in green. The last row reports the median results for all ECs.

	RF		XGB		Rocket		ANN		1D CNN		CNN		CNN Log	
	**Train**	**Test**	**Train**	**Test**	**Train**	**Test**	**Train**	**Test**	**Train**	**Test**	**Train**	**Test**	**Train**	**Test**
DCF	1	0.9	1	0.8833	1	1	1	1	1	1	1	0.9666	1	0.9666
FX	1	0.8333	1	0.8333	1	0.9666	0.9833	0.95	1	0.9333	1	0.9	1	0.8333
GLIPH	1	0.9666	1	0.9333	1	1	0.9833	0.9666	1	1	1	0.9333	1	0.9666
IBU	0.9916	0.8666	1	0.8333	0.9708	0.9	0.9916	0.95	1	0.9333	1	0.8666	1	0.833
PROP	1	0.9666	1	0.9666	1	1	1	0.9833	1	1	1	0.9666	1	1
SDS	1	0.8333	1	0.7916	1	0.9166	0.9895	0.9583	1	0.875	1	0.7916	1	0.75
TRI	1	0.9	1	0.8333	1	1	0.9833	0.9666	1	0.9666	1	0.9333	1	0.9333
Cu_d	1	0.8333	1	0.8888	1	1	0.9722	0.9444	1	0.9444	1	0.8333	1	0.8888
Cu_np	1	0.8888	1	0.8333	1	0.9444	0.9722	0.9444	1	0.9444	1	0.8888	1	0.8333
Ti_d	1	0.8888	1	0.8888	1	1	0.9861	1	1	1	1	0.8888	1	0.8611
Ti_np	1	0.9444	1	0.8888	1	1	0.9861	1	1	1	1	1	1	0.9444
Zn_d	1	0.8888	1	0.8888	1	0.9722	0.9861	0.9444	1	0.9444	1	0.8611	1	0.9444
Zn_np	0.9791	0.7777	1	0.7777	1	0.8888	0.9722	0.9166	1	0.8888	1	0.8611	1	0.7222
**Median**	1	0.8888	1	0.8833	1	1	0.9861	0.9583	1	0.9444	1	0.8888	1	0.8888

## Data Availability

The data presented in this study are available on request from the corresponding author. The data are not publicly available due to being part of an ongoing project.

## Appendix A

**Table A1:** Kruskal–Wallis *p*-values comparing all methods of the *EC* prediction task. Above the diagonal, we have the results for the test set, and below for the training set. Significant results (p<0.01) are represented in green or *red* when the method on the left is significantly better or worse, respectively.

EC	RF	XG	Rocket	ANN	1D CNN	CNN	CNN Log	
RF	―――	$1.23e^{-10}$	$2.58e^{-11}$	$2.80e^{-11}$	$1.89e^{-08}$	$2.63e^{-11}$	$2.68e^{-11}$	Test
XG	$2.24e^{-11}$	―――	$2.60e^{-11}$	$2.82e^{-11}$	$1.01e^{-07}$	$2.65e^{-11}$	$2.70e^{-11}$	
Rocket	$1.88e^{-11}$	$1.08e^{-05}$	―――	$2.59e^{-11}$	$4.04e^{-03}$	$3.64e^{-02}$	$5.79e^{-04}$	
ANN	$2.82e^{-11}$	$2.26e^{-11}$	$1.90e^{-11}$	―――	$7.04e^{-04}$	$2.64e^{-11}$	$2.69e^{-11}$	
1D CNN	$2.34e^{-11}$	$8.89e^{-02}$	$8.08e^{-01}$	$2.36e^{-11}$	―――	$3.19e^{-04}$	$2.68e^{-06}$	
CNN	$2.54e^{-11}$	$2.03e^{-11}$	$1.70e^{-11}$	$2.56e^{-11}$	$3.09e^{-08}$	―――	$6.93e^{-03}$	
CNN Log	$1.63e^{-09}$	$2.07e^{-11}$	$1.73e^{-11}$	$2.61e^{-11}$	$6.55e^{-09}$	$2.55e^{-02}$	―――	
Training								

**Table A2:** Kruskal–Wallis *p*-values comparing all methods of the *DCF* concentration prediction task. Above the diagonal, we have the results for the test set, and below for the training set. Significant results (p<0.01) are represented in green or *red* when the method on the left is significantly better or worse, respectively.

EC	RF	XG	Rocket	ANN	1D CNN	CNN	CNN Log	
RF	―――	$1.43e^{-01}$	$1.88e^{-11}$	$6.03e^{-11}$	$1.43e^{-04}$	$5.66e^{-07}$	$5.66e^{-07}$	Test
XG	$5.32e^{-03}$	―――	$1.40e^{-10}$	$1.89e^{-10}$	$2.98e^{-05}$	$1.73e^{-08}$	$1.73e^{-08}$	
Rocket	$5.32e^{-03}$	$1.00e^{+00}$	―――	$6.34e^{-01}$	$1.91e^{-03}$	$9.42e^{-08}$	$9.42e^{-08}$	
ANN	$2.98e^{-01}$	$1.36e^{-04}$	$1.36e^{-04}$	―――	$5.28e^{-03}$	$9.88e^{-07}$	$9.88e^{-07}$	
1D CNN	$9.28e^{-01}$	$5.34e^{-03}$	$5.34e^{-03}$	$2.08e^{-01}$	―――	$2.92e^{-01}$	$2.92e^{-01}$	
CNN	$5.32e^{-03}$	$1.00e^{+00}$	$1.00e^{+00}$	$1.36e^{-04}$	$5.34e^{-03}$	―――	$1.00e^{+00}$	
CNN Log	$5.32e^{-03}$	$1.00e^{+00}$	$1.00e^{+00}$	$1.36e^{-04}$	$5.34e^{-03}$	$1.00e^{+00}$	―――	
Training								

**Table A3:** Kruskal–Wallis *p*-values comparing all methods of the *FX* concentration prediction task. Above the diagonal, we have the results for the test set, and below for the training set. Significant results (p<0.01) are represented in green or *red* when the method on the left is significantly better or worse, respectively.

FX	RF	XG	Rocket	ANN	1D CNN	CNN	CNN Log	
RF	―――	$3.69e^{-01}$	$2.64e^{-06}$	$1.65e^{-06}$	$1.15e^{-02}$	$1.94e^{-02}$	$2.44e^{-01}$	Test
XG	$4.97e^{-01}$	―――	$1.03e^{-08}$	$2.31e^{-09}$	$1.00e^{-04}$	$7.84e^{-04}$	$7.65e^{-01}$	
Rocket	$6.16e^{-05}$	$5.92e^{-04}$	―――	$8.63e^{-01}$	$8.09e^{-03}$	$4.35e^{-07}$	$1.26e^{-09}$	
ANN	$6.54e^{-03}$	$7.45e^{-06}$	$4.94e^{-10}$	―――	$7.98e^{-03}$	$2.64e^{-07}$	$1.78e^{-10}$	
1D CNN	$4.13e^{-03}$	$4.52e^{-02}$	$4.02e^{-02}$	$3.34e^{-08}$	―――	$2.77e^{-02}$	$2.41e^{-05}$	
CNN	$6.16e^{-05}$	$5.92e^{-04}$	$1.00e^{+00}$	$4.94e^{-10}$	$4.02e^{-02}$	―――	$6.19e^{-08}$	
CNN Log	$6.16e^{-05}$	$5.92e^{-04}$	$1.00e^{+00}$	$4.94e^{-10}$	$4.02e^{-02}$	$1.00e^{+00}$	―――	
Training								

**Table A4:** Kruskal–Wallis *p*-values comparing all methods of the *GLIPH* concentration prediction task. Above the diagonal, we have the results for the test set, and below for the training set. Significant results (p<0.01) are represented in green or *red* when the method on the left is significantly better or worse, respectively.

GLIPH	RF	XG	Rocket	ANN	1D CNN	CNN	CNN Log	
RF	―――	$2.84e^{-03}$	$7.00e^{-06}$	$8.98e^{-01}$	$1.35e^{-02}$	$8.08e^{-04}$	$3.23e^{-01}$	Test
XG	$1.27e^{-03}$	―――	$2.80e^{-09}$	$7.73e^{-03}$	$3.12e^{-06}$	$8.80e^{-01}$	$6.39e^{-05}$	
Rocket	$1.27e^{-03}$	$1.00e^{+00}$	―――	$3.38e^{-04}$	$6.72e^{-02}$	$3.69e^{-11}$	$1.41e^{-04}$	
ANN	$2.65e^{-05}$	$5.40e^{-10}$	$5.40e^{-10}$	―――	$5.21e^{-02}$	$1.81e^{-04}$	$2.52e^{-01}$	
1D CNN	$8.34e^{-03}$	$3.17e^{-01}$	$3.17e^{-01}$	$1.13e^{-08}$	―――	$5.40e^{-09}$	$2.08e^{-01}$	
CNN	$1.27e^{-03}$	$1.00e^{+00}$	$1.00e^{+00}$	$5.40e^{-10}$	$3.17e^{-01}$	―――	$2.49e^{-10}$	
CNN Log	$1.27e^{-03}$	$1.00e^{+00}$	$1.00e^{+00}$	$5.40e^{-10}$	$3.17e^{-01}$	$1.00e^{+00}$	―――	
Training								

**Table A5:** Kruskal–Wallis *p*-values comparing all methods of the *IBU* concentration prediction task. Above the diagonal, we have the results for the test set, and below for the training set. Significant results (p<0.01) are represented in green or *red* when the method on the left is significantly better or worse, respectively.

IBU	RF	XG	Rocket	ANN	1D CNN	CNN	CNN Log	
RF	―――	$4.60e^{-03}$	$1.93e^{-01}$	$5.17e^{-04}$	$1.87e^{-03}$	$9.16e^{-01}$	$5.92e^{-01}$	Test
XG	$4.27e^{-06}$	―――	$2.63e^{-05}$	$1.24e^{-08}$	$5.16e^{-08}$	$4.61e^{-05}$	$3.68e^{-05}$	
Rocket	$3.07e^{-04}$	$4.38e^{-08}$	―――	$1.40e^{-02}$	$3.97e^{-02}$	$6.04e^{-03}$	$7.58e^{-02}$	
ANN	$9.75e^{-01}$	$2.88e^{-07}$	$1.11e^{-03}$	―――	$3.54e^{-01}$	$1.50e^{-08}$	$5.54e^{-06}$	
1D CNN	$1.09e^{-03}$	$4.02e^{-02}$	$1.08e^{-06}$	$2.07e^{-04}$	―――	$1.88e^{-07}$	$7.81e^{-05}$	
CNN	$4.27e^{-06}$	$1.00e^{+00}$	$4.38e^{-08}$	$2.88e^{-07}$	$4.02e^{-02}$	―――	$6.10e^{-02}$	
CNN Log	$4.27e^{-06}$	$1.00e^{+00}$	$4.38e^{-08}$	$2.88e^{-07}$	$4.02e^{-02}$	$1.00e^{+00}$	―――	
Training								

**Table A6:** Kruskal–Wallis *p*-values comparing all methods of the *PROP* concentration prediction task. Above the diagonal, we have the results for the test set, and below for the training set. Significant results (p<0.01) are represented in green or *red* when the method on the left is significantly better or worse, respectively.

PROP	RF	XG	Rocket	ANN	1D CNN	CNN	CNN Log	
RF	―――	$3.27e^{-01}$	$4.22e^{-05}$	$4.16e^{-02}$	$2.46e^{-03}$	$6.34e^{-01}$	$1.63e^{-06}$	Test
XG	$5.32e^{-03}$	―――	$1.09e^{-03}$	$3.62e^{-01}$	$4.54e^{-02}$	$1.71e^{-02}$	$5.79e^{-05}$	
Rocket	$5.32e^{-03}$	$1.00e^{+00}$	―――	$1.83e^{-02}$	$2.16e^{-01}$	$2.18e^{-10}$	$2.71e^{-01}$	
ANN	$9.69e^{-01}$	$2.65e^{-03}$	$2.65e^{-03}$	―――	$3.78e^{-01}$	$2.79e^{-03}$	$1.72e^{-03}$	
1D CNN	$2.83e^{-01}$	$4.02e^{-02}$	$4.02e^{-02}$	$2.19e^{-01}$	―――	$1.03e^{-04}$	$2.72e^{-02}$	
CNN	$5.32e^{-03}$	$1.00e^{+00}$	$1.00e^{+00}$	$2.65e^{-03}$	$4.02e^{-02}$	―――	$1.86e^{-11}$	
CNN Log	$5.32e^{-03}$	$1.00e^{+00}$	$1.00e^{+00}$	$2.65e^{-03}$	$4.02e^{-02}$	$1.00e^{+00}$	―――	
	Training							

**Table A7:** Kruskal–Wallis *p*-values comparing all methods of the *SDS* concentration prediction task. Above the diagonal, we have the results for the test set, and below for the training set. Significant results (p<0.01) are represented in green or *red* when the method on the left is significantly better or worse, respectively.

SDS	RF	XG	Rocket	ANN	1D CNN	CNN	CNN Log	
RF	―――	$1.89e^{-03}$	$4.23e^{-05}$	$1.76e^{-08}$	$9.15e^{-02}$	$1.17e^{-01}$	$4.13e^{-05}$	Test
XG	$6.28e^{-04}$	―――	$1.46e^{-09}$	$8.01e^{-11}$	$2.18e^{-05}$	$2.25e^{-02}$	$9.10e^{-01}$	
Rocket	$6.28e^{-04}$	$1.00e^{+00}$	―――	$3.35e^{-03}$	$1.46e^{-02}$	$4.23e^{-10}$	$1.40e^{-11}$	
ANN	$2.38e^{-04}$	$5.00e^{-09}$	$5.00e^{-09}$	―――	$5.19e^{-06}$	$5.93e^{-11}$	$1.40e^{-11}$	
1D CNN	$1.49e^{-02}$	$1.54e^{-01}$	$1.54e^{-01}$	$2.66e^{-07}$	―――	$6.22e^{-04}$	$2.38e^{-08}$	
CNN	$6.28e^{-04}$	$1.00e^{+00}$	$1.00e^{+00}$	$5.00e^{-09}$	$1.54e^{-01}$	―――	$2.62e^{-05}$	
CNN Log	$6.28e^{-04}$	$1.00e^{+00}$	$1.00e^{+00}$	$5.00e^{-09}$	$1.54e^{-01}$	$1.00e^{+00}$	―――	
	Training							

**Table A8:** Kruskal–Wallis *p*-values comparing all methods of the *TRI* concentration prediction task. Above the diagonal, we have the results for the test set, and below for the training set. Significant results (p<0.01) are represented in green or *red* when the method on the left is significantly better or worse, respectively.

TRI	RF	XG	Rocket	ANN	1D CNN	CNN	CNN Log	
RF	―――	$7.03e^{-04}$	$9.62e^{-11}$	$4.89e^{-08}$	$4.99e^{-07}$	$4.22e^{-03}$	$6.36e^{-02}$	Test
XG	$1.05e^{-02}$	―――	$1.67e^{-11}$	$1.05e^{-10}$	$1.32e^{-09}$	$3.22e^{-08}$	$7.14e^{-07}$	
Rocket	$1.05e^{-02}$	$1.00e^{+00}$	―――	$1.96e^{-03}$	$6.46e^{-04}$	$2.89e^{-12}$	$6.91e^{-12}$	
ANN	$8.89e^{-06}$	$1.59e^{-09}$	$1.59e^{-09}$	―――	$5.67e^{-01}$	$5.66e^{-08}$	$6.83e^{-07}$	
1D CNN	$8.16e^{-01}$	$1.06e^{-02}$	$1.06e^{-02}$	$2.67e^{-04}$	―――	$1.65e^{-09}$	$3.99e^{-08}$	
CNN	$1.05e^{-02}$	$1.00e^{+00}$	$1.00e^{+00}$	$1.59e^{-09}$	$1.06e^{-02}$	―――	$7.93e^{-01}$	
CNN Log	$1.05e^{-02}$	$1.00e^{+00}$	$1.00e^{+00}$	$1.59e^{-09}$	$1.06e^{-02}$	$1.00e^{+00}$	―――	
	Training							

**Table A9:** Kruskal–Wallis *p*-values comparing all methods of the *Cu_d* concentration prediction task. Above the diagonal, we have the results for the test set, and below for the training set. Significant results (p<0.01) are represented in green or *red* when the method on the left is significantly better or worse, respectively.

Cu_d	RF	XG	Rocket	ANN	1D CNN	CNN	CNN Log	
RF	―――	$5.93e^{-01}$	$6.08e^{-10}$	$1.60e^{-03}$	$4.64e^{-02}$	$1.19e^{-01}$	$2.90e^{-01}$	Test
XG	$5.97e^{-05}$	―――	$4.43e^{-11}$	$1.53e^{-03}$	$6.12e^{-02}$	$1.63e^{-03}$	$6.50e^{-01}$	
Rocket	$5.97e^{-05}$	$1.00e^{+00}$	―――	$3.66e^{-07}$	$1.26e^{-10}$	$1.54e^{-12}$	$1.01e^{-12}$	
ANN	$6.94e^{-09}$	$1.22e^{-11}$	$1.22e^{-11}$	―――	$7.96e^{-02}$	$6.24e^{-11}$	$1.69e^{-08}$	
1D CNN	$5.17e^{-04}$	$3.17e^{-01}$	$3.17e^{-01}$	$2.85e^{-10}$	―――	$1.94e^{-09}$	$2.36e^{-06}$	
CNN	$5.97e^{-05}$	$1.00e^{+00}$	$1.00e^{+00}$	$1.22e^{-11}$	$3.17e^{-01}$	―――	$7.24e^{-06}$	
CNN Log	$5.97e^{-05}$	$1.00e^{+00}$	$1.00e^{+00}$	$1.22e^{-11}$	$3.17e^{-01}$	$1.00e^{+00}$	―――	
	Training							

**Table A10:** Kruskal–Wallis *p*-values comparing all methods of the *Cu_np* concentration prediction task. Above the diagonal, we have the results for the test set, and below for the training set. Significant results (p<0.01) are represented in green or *red* when the method on the left is significantly better or worse, respectively.

Cu_np	RF	XG	Rocket	ANN	1D CNN	CNN	CNN Log	
RF	―――	$2.12e^{-02}$	$3.28e^{-06}$	$3.73e^{-05}$	$3.82e^{-04}$	$6.96e^{-01}$	$3.70e^{-02}$	Test
XG	$2.06e^{-02}$	―――	$1.20e^{-08}$	$3.32e^{-08}$	$2.82e^{-07}$	$2.64e^{-03}$	$3.08e^{-01}$	
Rocket	$2.06e^{-02}$	$1.00e^{+00}$	―――	$2.68e^{-01}$	$2.80e^{-02}$	$1.15e^{-07}$	$9.62e^{-10}$	
ANN	$4.23e^{-09}$	$1.40e^{-10}$	$1.40e^{-10}$	―――	$4.85e^{-01}$	$1.91e^{-07}$	$2.78e^{-09}$	
1D CNN	$8.49e^{-01}$	$4.02e^{-02}$	$4.02e^{-02}$	$4.21e^{-08}$	―――	$2.90e^{-07}$	$3.52e^{-09}$	
CNN	$2.06e^{-02}$	$1.00e^{+00}$	$1.00e^{+00}$	$1.40e^{-10}$	$4.02e^{-02}$	―――	$7.65e^{-03}$	
CNN Log	$2.06e^{-02}$	$1.00e^{+00}$	$1.00e^{+00}$	$1.40e^{-10}$	$4.02e^{-02}$	$1.00e^{+00}$	―――	
	Training							

**Table A11:** Kruskal–Wallis *p*-values comparing all methods of the *Ti_d* concentration prediction task. Above the diagonal, we have the results for the test set, and below for the training set. Significant results (p<0.01) are represented in green or *red* when the method on the left is significantly better or worse, respectively.

Ti_d	RF	XG	Rocket	ANN	1D CNN	CNN	CNN Log	
RF	―――	$8.88e^{-01}$	$6.10e^{-07}$	$3.86e^{-07}$	$2.36e^{-05}$	$1.72e^{-01}$	$1.16e^{-02}$	Test
XG	$1.27e^{-03}$	―――	$1.66e^{-06}$	$8.50e^{-07}$	$3.70e^{-05}$	$1.73e^{-01}$	$3.76e^{-02}$	
Rocket	$1.27e^{-03}$	$1.00e^{+00}$	―――	$6.24e^{-01}$	$6.19e^{-01}$	$3.88e^{-08}$	$5.14e^{-09}$	
ANN	$7.26e^{-04}$	$7.92e^{-07}$	$7.92e^{-07}$	―――	$2.06e^{-01}$	$5.14e^{-10}$	$1.10e^{-10}$	
1D CNN	$4.07e^{-01}$	$2.06e^{-02}$	$2.06e^{-02}$	$4.20e^{-04}$	―――	$1.23e^{-05}$	$2.54e^{-08}$	
CNN	$1.27e^{-03}$	$1.00e^{+00}$	$1.00e^{+00}$	$7.92e^{-07}$	$2.06e^{-02}$	―――	$2.53e^{-05}$	
CNN Log	$1.27e^{-03}$	$1.00e^{+00}$	$1.00e^{+00}$	$7.92e^{-07}$	$2.06e^{-02}$	$1.00e^{+00}$	―――	
	Training							

**Table A12:** Kruskal–Wallis *p*-values comparing all methods of the *Ti_np* concentration prediction task. Above the diagonal, we have the results for the test set, and below for the training set. Significant results (p<0.01) are represented in green or *red* when the method on the left is significantly better or worse, respectively.

Ti_np	RF	XG	Rocket	ANN	1D CNN	CNN	CNN Log	
RF	―――	$8.23e^{-04}$	$1.12e^{-04}$	$1.47e^{-03}$	$2.21e^{-02}$	$4.84e^{-06}$	$4.86e^{-01}$	Test
XG	$1.54e^{-01}$	―――	$8.15e^{-10}$	$1.51e^{-08}$	$4.89e^{-07}$	$4.45e^{-11}$	$5.04e^{-03}$	
Rocket	$1.54e^{-01}$	$1.00e^{+00}$	―――	$3.94e^{-01}$	$7.37e^{-02}$	$1.54e^{-01}$	$7.93e^{-06}$	
ANN	$1.86e^{-06}$	$3.06e^{-07}$	$3.06e^{-07}$	―――	$3.61e^{-01}$	$4.02e^{-02}$	$1.09e^{-04}$	
1D CNN	$5.84e^{-01}$	$3.17e^{-01}$	$3.17e^{-01}$	$1.15e^{-06}$	―――	$5.25e^{-03}$	$9.09e^{-04}$	
CNN	$1.54e^{-01}$	$1.00e^{+00}$	$1.00e^{+00}$	$3.06e^{-07}$	$3.17e^{-01}$	―――	$2.52e^{-07}$	
CNN Log	$1.54e^{-01}$	$1.00e^{+00}$	$1.00e^{+00}$	$3.06e^{-07}$	$3.17e^{-01}$	$1.00e^{+00}$	―――	
	Training							

**Table A13:** Kruskal–Wallis *p*-values comparing all methods of the *Zn_d* concentration prediction task. Above the diagonal, we have the results for the test set, and below for the training set. Significant results (p<0.01) are represented in green or *red* when the method on the left is significantly better or worse, respectively.

Zn_d	RF	XG	Rocket	ANN	1D CNN	CNN	CNN Log	
RF	―――	$9.88e^{-01}$	$9.85e^{-06}$	$4.13e^{-05}$	$1.13e^{-03}$	$1.00e^{+00}$	$1.90e^{-02}$	Test
XG	$5.34e^{-03}$	―――	$1.35e^{-07}$	$1.07e^{-06}$	$1.37e^{-04}$	$8.21e^{-01}$	$1.93e^{-03}$	
Rocket	$5.34e^{-03}$	$1.00e^{+00}$	―――	$1.68e^{-01}$	$3.51e^{-02}$	$7.79e^{-10}$	$2.72e^{-08}$	
ANN	$9.01e^{-06}$	$4.58e^{-09}$	$4.58e^{-09}$	―――	$1.66e^{-01}$	$6.71e^{-11}$	$1.29e^{-05}$	
1D CNN	$8.65e^{-02}$	$1.54e^{-01}$	$1.54e^{-01}$	$1.29e^{-07}$	―――	$6.29e^{-07}$	$3.27e^{-02}$	
CNN	$5.34e^{-03}$	$1.00e^{+00}$	$1.00e^{+00}$	$4.58e^{-09}$	$1.54e^{-01}$	―――	$3.28e^{-07}$	
CNN Log	$5.34e^{-03}$	$1.00e^{+00}$	$1.00e^{+00}$	$4.58e^{-09}$	$1.54e^{-01}$	$1.00e^{+00}$	―――	
Training								

**Table A14:** Kruskal–Wallis *p*-values comparing all methods of the *zn_np* concentration prediction task. Above the diagonal, we have the results for the test set, and below for the training set. Significant results (p<0.01) are represented in green or *red* when the method on the left is significantly better or worse, respectively.

Zn_np	RF	XG	Rocket	ANN	1D CNN	CNN	CNN Log	
RF	―――	$8.92e^{-01}$	$1.75e^{-06}$	$1.07e^{-05}$	$1.83e^{-03}$	$3.11e^{-03}$	$1.25e^{-01}$	Test
XG	$5.07e^{-06}$	―――	$7.84e^{-08}$	$1.63e^{-07}$	$6.85e^{-05}$	$2.59e^{-05}$	$4.68e^{-02}$	
Rocket	$1.94e^{-05}$	$3.17e^{-01}$	―――	$8.92e^{-01}$	$1.72e^{-01}$	$6.42e^{-03}$	$1.89e^{-10}$	
ANN	$1.05e^{-01}$	$1.26e^{-11}$	$4.89e^{-11}$	―――	$1.03e^{-01}$	$6.66e^{-04}$	$1.31e^{-10}$	
1D CNN	$2.74e^{-04}$	$7.81e^{-02}$	$3.13e^{-01}$	$1.80e^{-09}$	―――	$1.12e^{-01}$	$1.31e^{-06}$	
CNN	$5.07e^{-06}$	$1.00e^{+00}$	$3.17e^{-01}$	$1.26e^{-11}$	$7.81e^{-02}$	―――	$1.91e^{-08}$	
CNN Log	$5.07e^{-06}$	$1.00e^{+00}$	$3.17e^{-01}$	$1.26e^{-11}$	$7.81e^{-02}$	$1.00e^{+00}$	―――	
	Training							

## References

[1] Gavrilescu M., Demnerová K., Aamand J., Agathos S., Fava F. (2015). Emerging pollutants in the environment: Present and future challenges in biomonitoring, ecological risks and bioremediation. New Biotechnol..

[2] Robin L., Georg H., Michael A., John B., Anders B., Jordi D., Ian D., Yuriy D., Anja D., Barak H. (2010). Marine Strategy Framework Directive Task Group 8 Report: Contaminants and Pollution Effects.

[3] CAS (2011). CAS Registry Keeps Pace with Rapid Growth of Chemical Research, Registers 60 Millionth Substance.

[4] Duarte B., Gameiro C., Matos A.R., Figueiredo A., Silva M.S., Cordeiro C., Caçador I., Reis-Santos P., Fonseca V., Cabrita M.T. (2021). First screening of biocides, persistent organic pollutants, pharmaceutical and personal care products in Antarctic phytoplankton from Deception Island by FT-ICR-MS. Chemosphere.

[5] Reis-Santos P., Pais M., Duarte B., Caçador I., Freitas A., Pouca A.S.V., Barbosa J., Leston S., Rosa J., Ramos F. (2018). Screening of human and veterinary pharmaceuticals in estuarine waters: A baseline assessment for the Tejo estuary. Mar. Pollut. Bull..

[6] Carvalho R.C.D., Feijão E., Matos A., Cabrita M.T., Novais S., Lemos M., Caçador I., Marques J.C., Reis-Santos P., Fonseca V. (2020). Glyphosate-Based Herbicide Toxicophenomics in Marine Diatoms: Impacts on Primary Production and Physiological Fitness. Appl. Sci..

[7] Feijão E., Cruz de Carvalho R., Duarte I.A., Matos A.R., Cabrita M.T., Novais S.C., Lemos M.F.L., Caçador I., Marques J.C., Reis-Santos P. (2020). Fluoxetine Arrests Growth of the Model Diatom Phaeodactylum tricornutum by Increasing Oxidative Stress and Altering Energetic and Lipid Metabolism. Front. Microbiol..

[8] Franzitta M., Feijão E., Cabrita M.T., Gameiro C., Matos A., Marques J.C., Goessling J.W., Reis-Santos P., Fonseca V., Pretti C. (2020). Toxicity Going Nano: Ionic Versus Engineered Cu Nanoparticles Impacts on the Physiological Fitness of the Model Diatom Phaeodactylum tricornutum. Front. Mar. Sci..

[9] Duarte B., Gameiro C., Utkin A.B., Matos A.R., Caçador I., Fonseca V., Cabrita M.T. (2021). A multivariate approach to chlorophyll *A* Fluoresc. Data Trace Elem. Ecotoxicological Trials Using A Model Mar. Diatom. Estuarine Coast. Shelf Sci..

[10] Duarte B., Durante L., Marques J.C., Reis-Santos P., Fonseca V.F., Caçador I. (2021). Development of a toxicophenomic index for trace element ecotoxicity tests using the halophyte Juncus acutus: Juncus-TOX. Ecol. Indic..

[11] Anjum N., Duarte B., Caçador I., Sleimi N., Duarte A., Pereira E. (2016). Biophysical and Biochemical Markers of Metal/Metalloid-Impacts in Salt Marsh Halophytes and Their Implications. Front. Environ. Sci..

[12] Duarte B., Pedro S., Marques J.C., Adão H., Caçador I. (2017). Zostera noltii development probing using chlorophyll a transient analysis (JIP-test) under field conditions: Integrating physiological insights into a photochemical stress index. Ecol. Indic..

[13] Santos D., Duarte B., Caçador I. (2014). Unveiling Zn hyperaccumulation in Juncus acutus: Implications on the electronic energy fluxes and on oxidative stress with emphasis on non-functional Zn-chlorophylls. J. Photochem. Photobiol. B Biol..

[14] Carvalho R.C.D., Feijão E., Kletschkus E., Marques J., Reis-Santos P., Fonseca V., Papenbrock J., Caçador I., Duarte B. (2020). Halophyte bio-optical phenotyping: A multivariate photochemical pressure index (Multi-PPI) to classify salt marsh anthropogenic pressures levels. Ecol. Indic..

[15] Cabrita M.T., Gameiro C., Utkin A., Duarte B., Caçador I., Cartaxana P. (2016). Photosynthetic pigment laser-induced fluorescence indicators for the detection of changes associated with trace element stress in the diatom model species Phaeodactylum tricornutum. Environ. Monit. Assess..

[16] Duarte B., Feijão E., Cruz de Carvalho R., Duarte I.A., Silva M., Matos A.R., Cabrita M.T., Novais S.C., Lemos M.F.L., Marques J.C. (2020). Effects of Propranolol on Growth, Lipids and Energy Metabolism and Oxidative Stress Response of Phaeodactylum tricornutum. Biology.

[17] Duarte B., Cabrita M.T., Vidal T., Pereira J., Pacheco M., Pereira P., Canário J., Gonçalves F., Matos A., Rosa R. (2018). Phytoplankton community-level bio-optical assessment in a naturally mercury contaminated Antarctic ecosystem (Deception Island). Mar. Environ. Res..

[18] Rodrigues N.M., Batista J.E., Trujillo L., Duarte B., Giacobini M., Vanneschi L., Silva S. (2021). Plotting time: On the usage of CNNs for time series classification. arXiv.

[19] Koh J.C.O., Spangenberg G., Kant S. (2020). Automated Machine Learning for High-Throughput Image-Based Plant Phenotyping. bioRxiv.

[20] Guillard R., Ryther J. (1962). Studies of marine planktonic diatoms. I. Cyclotella nana Hustedt, and Detonula confervacea (cleve) Gran. Can. J. Microbiol..

[21] Feijão E., Gameiro C., Franzitta M., Duarte B., Caçador I., Cabrita M.T., Matos A. (2018). Heat wave impacts on the model diatom Phaeodactylum tricornutum: Searching for photochemical and fatty acid biomarkers of thermal stress. Ecol. Indic..

[22] Cabrita M.T., Duarte B., Gameiro C., Godinho R., Caçador I. (2018). Photochemical features and trace element substituted chlorophylls as early detection biomarkers of metal exposure in the model diatom Phaeodactylum tricornutum. Ecol. Indic..

[23] Duarte B., Prata D., Matos A., Cabrita M.T., Caçador I., Marques J., Cabral H., Reis-Santos P., Fonseca V. (2019). Ecotoxicity of the lipid-lowering drug bezafibrate on the bioenergetics and lipid metabolism of the diatom Phaeodactylum tricornutum. Sci. Total Environ..

[24] Silva M., Feijão E., da Cruz de Carvalho R., Duarte I.A., Matos A., Cabrita M.T., Barreiro A., Lemos M., Novais S., Marques J.H. (2020). Comfortably numb: Ecotoxicity of the non-steroidal anti-inflammatory drug ibuprofen on Phaeodactylum tricornutum. Mar. Environ. Res..

[25] Duarte B., Marques J.C., Caçador I. (2015). Ecophysiological response of native and invasive Spartina species to extreme temperature events in Mediterranean marshes. Biol. Invasions.

[26] Breiman L. (2001). Random Forests. Mach. Learn..

[27] Chen T., Guestrin C. XGBoost: A Scalable Tree Boosting System. Proceedings of the 22nd ACM SIGKDD International Conference.

[28] Dempster A., Petitjean F., Webb G.I. (2020). ROCKET: Exceptionally fast and accurate time series classification using random convolutional kernels. Data Min. Knowl. Discov..

[29] Goodfellow I., Bengio Y., Courville A. (2016). Deep Learning.

[30] Pedregosa F., Varoquaux G., Gramfort A., Michel V., Thirion B., Grisel O., Blondel M., Prettenhofer P., Weiss R., Dubourg V. (2011). Scikit-learn: Machine Learning in Python. J. Mach. Learn. Res..

[31] Abadi M., Agarwal A., Barham P., Brevdo E., Chen Z., Citro C., Corrado G.S., Davis A., Dean J., Devin M. (2015). TensorFlow: Large-Scale Machine Learning on Heterogeneous Systems. tensorflow.org.

